# Factors associated with work-private life conflict and leadership qualities among line managers of health professionals in Swiss acute and rehabilitation hospitals – a cross-sectional study

**DOI:** 10.1186/s12913-021-06092-1

**Published:** 2021-01-22

**Authors:** Karin A. Peter, Ruud J. G. Halfens, Sabine Hahn, Jos M. G. A. Schols

**Affiliations:** 1 Division of Applied Research & Development in Nursing, Bern University of Applied Sciences, Bern, Switzerland; 2 Department of Health Services Research, CAPHRI - Care and Public Health Research Institute, Maastricht University, Maastricht, the Netherlands; 3 Department of Health Services Research -Focusing on Value-based Care and Ageing- and Department of Family Medicine, CAPHRI - Care and Public Health Research Institute, Maastricht University, Maastricht, the Netherlands

**Keywords:** Stressors, Work-related stressors, Health professionals, Hospital, Work-private life conflict, Quality of leadership

## Abstract

**Background:**

The workforce shortage of health professionals is a matter of global concern. Among possible causative factors in this shortage are the incompatibility of health professionals’ work with their private life, which may lead to increased stress and burnout symptoms, job dissatisfaction and a higher intention to leave the profession prematurely. Also, poor leadership qualities among direct line managers (e.g. clinic directors, leading physicians, ward managers, team leaders) have been associated with health professionals’ job dissatisfaction and intention to leave in previous studies. This study therefore aimed to identify key factors associated with health professionals’ work-private life conflicts and their managers’ leadership quality.

**Methods:**

The study is based on a cross-sectional survey in 26 Swiss acute and rehabilitation hospitals, consisting of 3398 health professionals from various disciplines. For data analysis, multilevel models (with hospitals as the second level variable) were performed for ‘work-private life conflict’ and ‘quality of leadership’, considering significant main effects (using AIC) and significant interactions (using BIC) of potential explanatory variables.

**Results:**

The main findings reveal that the compatibility of health professionals’ work and private life is associated with how much they can influence shift planning (possibility of exchanging shifts, B = -2.87, *p* < 0.01), the extent to which their individual preferences are considered (e.g. working on one specific shift only, B = 6.31, *p* < 0.01), number of work shifts per weekend (B = 1.38, *p* < 0.01) and working hours per week (B = 0.13, *p* < 0.01). In addition, the factors high quantitative demands (B = 0.25, *p* < 0.01), being required to hide their emotions (B = 0.16, *p* < 0.01) and poor social community support at work (B = -0.12, *p* < 0.01) were related to severe work-private life conflicts among health professionals. Regarding managerial leadership, health professionals perceived the leadership qualities of their direct line manager as being better if they received more social support (B = 0.61, *p* < 0.01) and rewards (B = 0.41, *p* < 0.01) at work.

**Conclusions:**

The results show key components of improving the compatibility of work and private life for health professionals as well as managerial leadership qualities, and may help leaders working in acute or rehabilitation hospitals to develop appropriate interventions.

## Background

Health systems around the globe are struggling due to insufficient availability of health professionals [1]. Not only are the demographic challenges associated with an ageing population relevant aspects of this workforce shortage, but also the occurrence of chronic diseases and the resulting high demand for treatment, many expected retirements of health professionals, lack of young talent and health professionals leaving their profession prematurely [1, 2]. Therefore, improving working conditions and reducing work-related stress is essential for keeping experienced health professionals in the health care system [3, 4]. Work-private life conflicts and poor managerial leadership qualities have been identified as two of the most important stressors and associated with health professionals’ dissatisfaction at work, with poor health-related outcomes and with more frequent intentions to leave the profession prematurely [5, 6]. In a previous study, these two stressors were found to be of common relevance for all health professionals (e.g. nurses, physicians, medical-therapeutic professionals) [6].

A work-private life conflict is described as a ‘conflict a person may experience between the work role and other life roles’ [7]. Most health professionals (e.g. nurses, midwives, physicians) are affected, since the 24-h operation mode requires staff presence at all times, especially in acute care hospitals. Work-private life conflicts are associated with increased behavioural and cognitive stress symptoms, job dissatisfaction, firmer intentions to leave the profession as well as increased burnout symptoms and poor quality of sleep [6, 8, 9]. Specific factors that have the potential to increase or decrease work-private life conflicts in the daily work of health professionals are related to topics of shift planning and employment status (e.g. a higher number of working hours, nonstandard working hours) or health professionals’ lack of influence over their work schedules [10]. However, to design effective mitigating strategies, it is important to identify further associated factors that can positively or negatively influence work-private life conflicts among health professionals.

Healthcare leaders also play an important role in shaping the working conditions, environment and compatibility between work and private life of the employees in their units [11]. Quality of leadership can best be described as the ‘extent to which leaders actively contribute to a positive work climate and clarity (e.g. of tasks and roles) and effort to achieve common goals’ [12]. A leader’s behaviour has the potential to either prevent or cause stress at work [13–15] and poor managerial leadership qualities have been associated with health professionals’ job dissatisfaction and more frequent intentions to leave their organisation and profession [6, 16]. Therefore, high quality leadership and management competencies are not only important in preventing and reducing stress at work but also in retaining health professionals in their work [11]. So far, important factors that are positively or negatively associated with managerial leadership qualities have been identified as leaders’ appropriate management of workload, resources and conflicts as well as their provision of social support and reward to employees [11, 17].

However, in order to develop strategies addressing work-private life conflicts effectively and enhance managerial leadership qualities in the healthcare sector, further in-depth knowledge about health professionals is important. In Switzerland, a full-time employee generally works on average slightly longer hours (42.5 h per week) compared to a full-time employee in the European Union (39.3 h per week) [18]. Therefore, it is important to identify factors that are positively or negatively associated with health professionals’ work-private life conflicts and their perception of managerial leadership qualities in order to design effective interventions to counteract the shortage of health professionals in the Swiss health care system.

## Method

### Aim of the study

The aim of this study was to identify key factors regarding health professionals’ demands at work, work organisation and job content, social relations and leadership, person-work interface factors, work-private life (im)balances, clinical work settings and employment, demographic or work schedule information (independent variables) that are associated with (1) work-private life conflicts and (2) the perceived quality of leadership (dependent variables) by those working in Swiss acute care and rehabilitation hospitals. The study also aimed to provide detailed information regarding different roles and professional groups within the healthcare professions, such as general registered nurses, assistant nurses, advanced practice nurses (APN) or clinical nurse specialists (CNS), midwives, physicians, medical-technical professionals (e.g. biochemical analysts, paramedics, specialists for medical-technical radiology) and medical-therapeutic professionals (e.g. physiotherapists, occupational therapists, dieticians).

### Design

The study is part of the national **STRAIN** study, i.e., ‘work-related **STR**ess **A**mong health professionals **IN** Switzerland’ (Clinical Trials registration: NCT03508596). The dataset for this study was collected between September 2017 and March 2018 under a cross-sectional design.

### Questionnaire

The STRAIN questionnaire was used, which is based on the model of ‘causes and consequences of work-related stress’ published in Eurofound [19] and consists of well established, valid and reliable scales from the Copenhagen Psychosocial Questionnaire - COPSOQ [20–22], the NEXT questionnaire [23], the Oslo social support scale (Oslo-3) [24, 25] and the Sixth European Working Conditions Survey - EWCS [26]. The COPSOQ is freely accessible. Written permission to use scales/items from the NEXT and the EWCS questionnaires as well as from the OSLO-3 scale was obtained for this study from the original authors.

**Fig. 1:**
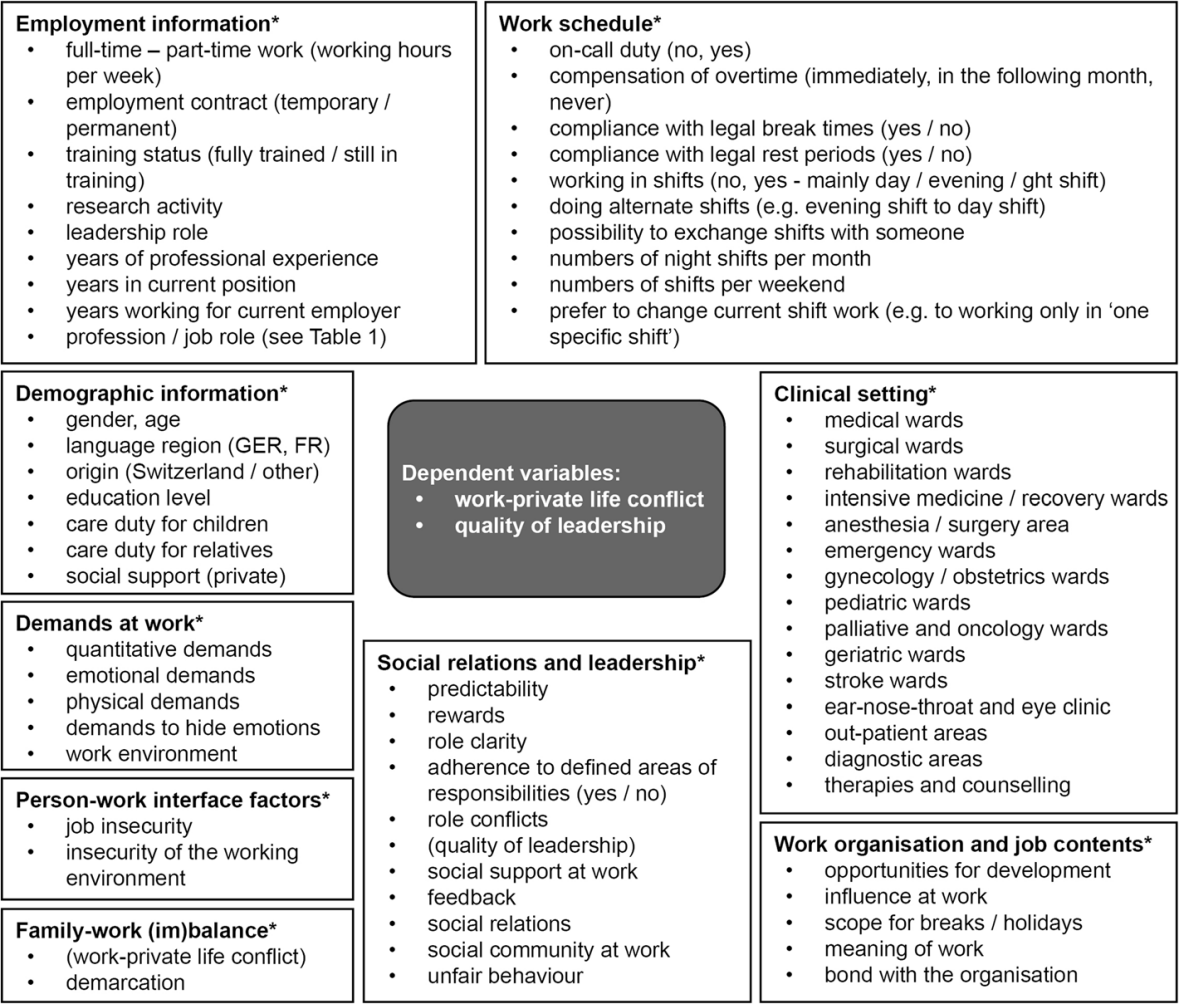
Analysis model for multiple linear regression models. *variables used as ‘independent variables’ in the regression models

The two scales of COPSOQ on ‘work-privacy-conflict’ (Cronbach’s alpha: 0.92 [27]) and ‘quality of leadership’ (Cronbach’s alpha: 0.89 [27]) were used as dependent variables (see Fig. 1).

The scale on ‘work-privacy-conflict’ consists of five items with 5-point Likert-type answer categories (to a very large extent, to a large extent, somewhat, to a small extent, to a very small extent) surveying the influence of work on the private life of employees, e.g. ‘the demands of my work interfere with my home, personal and family life’ or ‘due to work-related duties, I have to make changes in my plans for family or personal activities’.

The scale on ‘quality of leadership consists of four items with 5-point Likert-type answer categories (to a very large extent, to a large extent, somewhat, to a small extent, to a very small extent) asking employees about quality of leadership in terms of their immediate superior, e.g. ‘to what extent would you say that your superior makes sure that the individual member of the staff has good development opportunities?’, ‘gives high priority to job satisfaction?’, ‘is good at work planning?’ and ‘is good at solving conflicts?’

As independent variables, several scales and single items on ‘demographic’ and ‘employment’ information, health professionals’ ‘work schedule’, ‘clinical settings’ as well as various ‘demands at work’, ‘social relations and leadership’, ‘person-work interface factors’, ‘work organisation and content’ were used. The single items on ‘work schedule’ are based on the Swiss labour law for health professionals (legal break time in Switzerland is 15 min for 5.5 h, 30 min for 7–9 h and 60 min for more than 9 h working time, and a minimum of 9 h rest period between two shifts). More details on dependent and independent variables are presented in Fig. 1.

### Setting and data collection

The study was conducted in the Swiss healthcare system, which includes influences of direct democracy with aspects of managed competition and is highly complex. Despite its small population (8.6 million inhabitants), Switzerland has a total of 293 acute care, rehabilitation and psychiatric hospitals that can vary in size from 2 to 3 to more than 2′000 beds [28]. Since many of the Swiss acute care hospitals have integrated their rehabilitation wards internally, acute care and rehabilitation hospitals were combined for this study. Therefore, acute care and rehabilitation hospitals were randomly selected (using randomizer.org) from all registered hospitals in Switzerland from the Swiss Federal Statistical Office in 2016 between March 2017 and September 2017. Organisations which were too small (average number of beds < 20, less than 7 employees), or with a specialisation, e.g. in gynaecology or neonatology only were excluded. To ensure a sufficiently large study sample, 70 acute and rehabilitation hospitals were invited to participate. In total, 26 acute care and rehabilitation hospitals participated in this study. The acute care and rehabilitation hospitals included various medical, surgical, rehabilitation, geriatric, palliative, and paediatric wards, as well as gynaecology/obstetrics, anaesthesia, surgical, emergency and intensive medicine, diagnostic and out-patient areas.

In each participating hospital, a contact person was available who distributed the questionnaire to all health professionals, working within the organisation (e.g. nursing staff, midwives, physicians, medical-technical and medical-therapeutic professionals) at all skill levels. Participation in the study was voluntary for the organisation as well as for all health professionals. A written study flyer and a short film were used to inform participants about the aim and content of the study. Participants had between 4 and 6 weeks to fill out the online or paper questionnaire (between September 2017 and March 2018). They took 35 min on average to complete the total questionnaire.

### Data analysis

Data were analysed using R version 3.6.0. For data analysis, all Items assessing ‘demands at work’, ‘person-work interface factors’, ‘social relations and leadership’ (except the items on adherence to defined areas of responsibilities), ‘family-work (im)balance’ and ‘work organisation and job contents’ (see Fig. 1) were transformed and standardised on a value range from 0 to 100 points (0 was the minimum, 100 the maximum value), considering reverse scored items. If less than half of the questions in a scale had been answered, no average score was calculated [21]. Items/scales on ‘demographic information’, ‘employment information’, ‘work schedule’ and ‘clinical settings’ were excluded from this transformation.

First, data were analysed descriptively. Therefore, the mean and standard deviation of work stressors for various health professions were calculated and tested for significant differences between these professions. Since the test of homogeneity of variance was significant and there were no equal-sized samples of data, the Kruskal-Wallis test (Monte Carlo based on 10,000 samples, 99% confidence interval) was used to test for differences between professional groups regarding work stressors.

Second, we estimated a multilevel model for the two outcome variables ‘work-private life conflict’ and ‘quality of leadership’ with the 26 hospitals as the second level variable, and all independent variables (presented in Fig. 1) were used as potential explanatory variables on the level of individuals (first level). In order to take account of important interactions between explanatory variables in the analysis, two models were calculated for each outcome variable (step 1 and step 2). In step 1, from all independent variables available in the STRAIN questionnaire, we selected those variables with a significant main effect on the target variable in a linear regression model, using the stepwise backward algorithm (R-package MASS, function stepAIC) and the Akaike Information Criterion (AIC). In step 2, from the explanatory variables selected in step 1, we examined interactions of variable pairs (i.e. two-way interactions). Starting with the model from step 1, we used the stepwise both-direction algorithm and the Bayesian Information Criterion (BIC) to select the significant interactions and deselect insignificant main effects (using the less strict AIC had led to excessively large models with > 40 interactions).

Finally, we estimated a multilevel model with the 26 hospitals as the second level variable and the main and interaction effects found as fixed effects on the level of individuals (first level). We therefore used the bootstrap procedure to estimate the standard deviations and the *p*-values of the coefficients.

## Results

### Study sample

In total, 3398 health professionals working in 26 acute care or rehabilitation hospitals took part in the study, 80% from the German-speaking part and 20% from the French-speaking part of Switzerland, thus approximating national proportions (response rate between 11 and 73%). The study sample included 69% nurses, of whom 43.5% were general registered nurses, 9.6% nurses with additional training in emergency medicine / intensive care / anaesthesia, 14.6% nurse assistants and 1.5% advanced practice nurses (APN) or clinical nurse specialists (CNS). Also included in the study sample were 2% midwives, 11% physicians, 9% medical-technical professionals and 9% medical-therapeutic professionals. Most participants were female (81%) with a mean age of 40 years; further means were18 years of professional experience and 8 years working in their current position. The majority (67%) of participating health professionals originated from Switzerland or from Germany (13%).

### Results regarding different health professions

Table 1 presents an overview of the dependent and independent variables (scales only) of the mean, standard deviation and significant differences among various nursing positions (e.g. general registered nurses, assistant nurses, advanced practice nurse (APN) or clinical nurse specialist (CNS)), midwives, physicians, medical-technical professionals and medical-therapeutic professionals.

**Table 1:** mean, standard deviation and Kruskal-Wallis test on various health professions

	registered nurses (RN) (*n* = 1182)		RN with special training^a^ (*n* = 273)		nurse assistants (*n* = 430)		APN / CNS (*n* = 47)		Midwives (*n* = 63)		Physicians (*n* = 299)		medical-technical professionals (*n* = 241)		medical-therapeutic-professionals (*n* = 238)	
	Mean	SD	Mean	SD	Mean	SD	Mean	SD	Mean	SD	Mean	SD	Mean	SD	Mean	SD
**Dependent variables**																
Work-private life conflict ***	32.47	21.39	32.08	20.01	33.62	22.32	34.00	19.18	37.71	14.45	**50.20**	22.15	25.51	18.72	**23.01**	17.83
Quality of leadership*	62.71	22.87	59.17	22.26	63.94	22.69	63.32	23.74	**58.16**	19.60	60.37	23.65	60.54	23.82	**64.64**	21.65
**Independent variables**																
**Demands at work**																
Quantitative demands***	62.12	14.97	58.88	13.94	58.06	15.91	61.35	14.86	63.84	10.00	**67.15**	15.89	57.19	13.90	**56.57**	16.66
Emotional demands***	61.85	14.54	65.56	12.54	**66.10**	15.18	61.57	17.92	**43.95**	11.37	60.37	12.86	52.74	16.28	58.75	11.38
Physical demands***	43.52	21.65	44.27	19.56	**53.89**	22.40	39.53	23.24	33.89	15.93	**23.85**	14.77	40.76	19.89	34.57	21.96
Demands to hide emotions*	42.53	22.99	**47.24**	21.57	42.06	23.48	43.75	19.14	45.97	15.82	43.99	21.22	**42.29**	22.64	43.17	20.06
Work environment***	41.41	18.39	**51.03**	18.41	41.43	17.93	40.63	22.94	27.46	14.51	30.50	20.05	45.48	17.80	**26.33**	18.75
**Work organization and job contents**																
Possibilities for development***	73.60	14.17	71.62	14.53	**70.88**	16.69	71.81	16.90	76.21	12.61	**77.48**	14.61	73.37	14.15	75.07	13.99
Influence at work***	48.13	19.45	45.62	18.01	45.93	20.17	54.89	19.29	43.44	15.45	49.61	21.35	**40.60**	20.50	**59.67**	16.21
Scope for breaks / holidays***	57.15	19.68	57.60	18.71	**53.25**	21.40	62.77	23.20	54.17	19.07	60.34	18.33	**63.22**	16.55	55.21	20.86
Meaning of work***	84.21	15.53	82.53	15.94	81.85	16.74	**77.72**	16.86	**90.32**	12.69	82.47	18.18	83.97	16.24	79.73	15.67
Bond with the organization***	59.14	19.84	**54.37**	19.53	61.69	21.37	55.16	16.37	56.14	17.58	59.92	19.00	**63.79**	18.44	60.42	17.76
**Social relations and leadership**																
Predictability	62.23	19.28	60.20	18.57	64.34	19.49	61.14	18.30	**64.75**	15.89	**59.73**	19.91	63.01	17.93	61.99	18.78
Rewards*	53.54	26.62	49.91	24.91	53.12	27.66	54.89	27.19	**48.33**	23.41	**56.48**	27.86	55.68	25.32	52.27	26.18
Role clarity***	80.24	13.80	79.58	13.31	80.29	14.11	**74.46**	17.07	77.73	11.88	76.36	15.33	**80.30**	14.60	75.69	14.50
Role conflicts***	41.49	20.41	42.02	20.04	41.17	21.45	46.20	22.20	**49.18**	17.92	41.25	20.31	36.14	19.45	**35.29**	18.27
Social support at work*	75.23	16.79	73.77	15.61	73.47	17.08	74.32	17.59	73.62	15.66	**72.54**	20.52	75.53	16.06	**78.21**	16.69
Feedback***	50.03	20.08	**44.15**	19.65	**53.48**	20.64	50.82	18.71	50.64	17.28	44.60	21.79	47.39	20.66	45.87	19.96
Social relations***	62.45	22.81	62.19	24.72	66.34	22.24	61.67	25.89	55.51	23.24	63.70	23.99	**72.26**	22.42	**52.31**	26.97
Social community at work***	78.33	13.72	**75.94**	11.82	78.42	15.36	78.53	14.83	77.33	13.23	79.50	14.29	79.82	12.86	**83.47**	14.72
Unfair behavior***	14.47	21.27	14.16	19.81	18.48	24.03	**22.22**	25.13	18.97	23.09	13.30	20.76	15.55	21.58	**9.65**	17.34
**Person-work interface factors**																
Job insecurity***	16.28	17.94	14.00	16.64	23.55	21.33	20.70	23.29	16.33	19.47	**13.20**	15.69	**21.61**	19.89	15.03	15.34
Insecurity of work environment***	31.94	25.05	34.84	26.30	**37.84**	26.84	36.41	28.12	29.71	23.40	32.28	24.76	33.57	23.04	**27.91**	22.55
**Work-private life (im)balance**																
Demarcation***	33.58	21.27	34.99	22.00	36.39	22.09	35.83	21.25	35.99	19.74	**49.63**	25.09	36.93	22.33	**31.89**	21.66

highest and lowest mean are marked; significant Kruskal-Wallis Test *p < 0.05, **p < 0.01, ***p < 0.001, ^a^RN with special training in emergency medicine, intensive care or anaesthesia

The highest mean for the scales on ‘demands at work’ was found among physicians for high ‘quantitative demands’ (e.g. work at a high pace, doing overtime) *(mean = 67.2, SD = 15.9)* and among nurse assistants for high ‘emotional demands’ at work (e.g. confrontation with death, suffering, aggressive patients) *(mean = 66.1, SD = 15.2).* Regarding ‘work organisation and job content’, the lowest mean was revealed for the scale on ‘influence at work’ (e.g. degree of influence concerning work, amount of work, what to do) among medical-technical professionals *(mean = 40–6, SD = 20.5)* as well as on ‘scope for breaks and holidays’ among nurse assistants *(mean = 53.4, SD = 21.4).* For ‘social relations and leadership’ the lowest mean was found for the scale on ‘feedback’ received from their line manager among registered nurses with training in emergency medicine, intensive care or anaesthesia *(mean = 44.2, SD = 19.7).* Regarding ‘person-work interface factors’ the highest mean was reached by the scale on ‘insecurity of the working environment’ (e.g. unforeseen changes in shift schedules, working times) among nurse assistants *(mean = 37.8, SD = 26.8)*. For the scale on ‘work-private life conflict’ as well as difficulties with ‘demarcation’ (e.g. being available in leisure time for work issues) the highest mean was found among physicians *(work-private life conflict: mean = 50.2, SD = 22.2; demarcation: mean = 49.6, SD = 25.1).*

### Descriptive results on overtime, break times, rest periods and shift work

Descriptive results revealed that 63% of the physicians and 30% of the nurses and midwives have to do overtime ‘often’ to ‘always’ (presented in Table 2). In addition, 35% of all physicians and 6% of all medical-therapeutic professionals stated that they have no means to record their overtime at work. Furthermore, 53% of physicians, 9% of medical-technical professionals and 7% of nurses, midwives and medical-therapeutic professionals stated that it is impossible to be compensated for working overtime (either by time off or supplementary payment).

**Table 2:** Descriptive results on overtime, compliance with break times / rest periods and shift work, duty planning and satisfaction with shift work

	nurses & midwives	physicians	medical-technical prof.	medical-therapeutic prof.
	*n = 1864*	*n = 284*	*n = 207*	*n = 230*
**Doing overtime**				
Often-always	30%	63%	26%	25%
Sometimes	47%	24%	54%	54%
Seldom-never	23%	12%	20%	22%
**Assessment of overtime**				
Can count overtime	95%	57%	95%	93%
Cannot measure overtime	2%	35%	1%	6%
Can measure their overtime, but do not do it	3%	8%	3%	2%
**Compensation for overtime** (multiple responses)				
Compensation for overtime in the same month by holidays or free time	22%	25%	28%	54%
Compensation for overtime in the following month or later by holidays or free time	86%	57%	85%	81%
Not possible to compensate for overtime at all	7%	53%	9%	7%
Compensation by getting paid for overtime	15%	16%	22%	8%
**Compliance with break times**				
Break times often-always take place	65%	50%	72%	82%
Break times sometimes take place	22%	22%	21%	11%
Break times seldom-never take place	13%	28%	7%	8%
**Compliance with rest periods**				
Rest periods are often-always observed	80%	62%	82%	93%
Rest periods are sometimes observed	14%	28%	13%	4%
Rest periods are seldom-never observed	6%	10%	5%	3%
**Working in shifts** (filter question, if ‘yes’ further questions)				
Yes	96%	90%	96%	18%
No	4%	10%	4%	82%
**Influence on their duty scheduling**	*n = 1511*	*n = 127*	*n = 172*	*n = 14*
‘Some - no’ influence on duty scheduling	84%	73%	71%	84%
‘Great’ influence on duty scheduling	16%	27%	29%	16%
**Preference to change current shift work**				
Yes	50%	47%	27%	50%
No	50%	53%	73%	50%
**Satisfaction with shift work**				
Not satisfied with shift work in terms of their private life	21%	36%	11%	9%
Not satisfied with shift work in terms of their personal well-being	33%	54%	17%	15%

*n* number of cases

Descriptive findings on compliance with legal break times reveal that 28% of the physicians, 13% of the nurses/midwives, 7% of the medical-technical and 8% of the medical-therapeutic professionals stated that their break times seldom to never take place. Moreover, 10% of the physicians and 6% of the nurses and midwives reported that legal rest periods are seldom to never observed.

In total, 96% of the nurses and midwives, 90% of the physicians, 96% of the medical-technical and 18% of the medical-therapeutic professionals stated that they worked in shifts, with most of them having a restricted amount or no influence on their duty scheduling. Of these health professionals working in shifts, 50% of the nurses, midwives and physicians stated that they would change their current shift work (e.g. to working only ‘one specific shift’) if they could. Additional findings on satisfaction regarding their shift work reveal that 36% of the physicians and 21% of the nurses/midwives are not satisfied with their working hours in terms of their personal well-being. Moreover, 54% of the physicians, 33% of the nurses/midwives, 17% of the medical-technical and 15% of the medical-therapeutic professionals are not satisfied with their working hours regarding the compatibility between work and private life.

Results for the final multilevel model on the work-private life conflict are presented in Table 3 (predictors explained 48.8% of the variance). The topics shift work and influence on work schedule were found to be the strongest predictors for a severe work-private life conflict among health professionals. The results indicate that health professionals’ preference to change their current shift work (e.g. to work one specific shift only) was strongly related to a work-private life conflict *(B = 6.31, p = 0.000).* A further strong predictor of a work-private life conflict was if health professionals stated that they were not able to exchange shifts with other team members *(B = -2.87, p = 0.002)*. An increasing number of shifts per weekend was also a predictor of a severe work-private life conflict *(B = 1.38, p = 0.002)* among health professionals. In addition, a lower ‘scope for breaks and holidays’ was also determined to be related to a severe work-private life conflict *(B = − 0.07, p = 0.000).*

**Table 3:** Results of multiple regression analysis on ‘work-private life conflict

coefficients	estimate (B)	std. estimate (*β*)	std. error^a^	t-value^a^	*p*-value^a^
(Intercept)	10.83	0.00	4.42	2.45	0.008
Quantitative demands	0.25***	0.18	0.04	5.93	0.000
Role conflicts	0.09***	0.08	0.02	4.16	0.000
Demands to hide emotions	0.16***	0.16	0.02	6.87	0.000
Scope for breaks / holidays	-0.07***	−0.06	0.02	−3.78	0.000
Meaning of work	−0.10***	−0.07	0.02	−4.18	0.000
Bond with the company	−0.08***	− 0.07	0.02	−3.92	0.000
Social community at work	−0.12***	−0.08	0.03	−4.43	0.000
Insecurity of the working environment	0.10***	0.12	0.02	6.53	0.000
Demarcation	−0.06	− 0.06	0.06	− 0.89	0.368
Full-time – part-time work (working hours per week)	0.13***	0.12	0.02	6.53	0.000
Years working in current position	0.13	0.05	0.10	1.34	0.190
Possibility to exchange shifts (1 = yes, 0 = no)	−2.87**	−0.06	0.82	−3.49	0.002
Would change their current shift work (e.g. to working only in ‘one specific shift’) (1 = yes, 0 = no)	6.31***	0.14	0.84	7.50	0.000
Number of shifts per weekend	1.38**	0.07	0.42	3.26	0.002
Care tasks for children privately (1 = yes, 0 = no)	3.76***	0.09	0.77	4.88	0.000
Profession: physician	12.23***	0.14	1.47	8.33	0.000
Profession: administration & research	−5.90**	−0.05	1.93	−3.06	0.006
Interactions – demands to hide emotions & years working in current position	−0.01***	− 0.14	0.00	−3.44	0.000
Interactions – quantitative demands & demarcation	0.00***	0.24	0.00	3.25	0.000

^a^based on bootstrap, *p < 0.05, **p < 0.01, ***p < 0.001

Further results on employment status indicated that an increasing number of working hours per week (working full time) predicted a severe work-private life conflict *(B = 0.13, p = 0.000)*. In addition, private care duties with children also appeared to be a predictor of a severe work-private life conflict *(B = 3.76, p = 0.000)*.

Other results show that higher ‘quantitative demands’ at work *(B = 0.25, p = 0.000)*, higher ‘demands for hiding emotions’ (e.g., hiding feelings) *(B = 0.16, p = 0.000)* as well as a lower perception of ‘social community at work’ (e.g. atmosphere and co-operation between colleagues) *(B = -0.12, p = 0.000)* were also associated with a severe work-private life conflict among health professionals. In addition, existing ‘role conflicts’ among health professionals due to contradictory role requirements at work, was also identified as a predictor for a work-private life conflict *(B = 0.09, p = 0.000)*. More results on work-organisation and content also revealed a lower ‘meaning of work’ (e.g. perceive work as meaningful / important) *(B = -0.10, p = 0.000)* and ‘bond with the organisation’ *(B = -0.08, p = 0.000)* as well as a higher ‘insecurity of the working environment’ *(B = 0.10, p = 0.000)* as being related to a severe work-private life conflict among health professionals.

When the different health professions are compared, physicians seem to have a more severe *(B = 12.23, p = 0.000)* and employees working in the field of administration and research a less pronounced work-private life conflict *(B = -5.90, p = 0.006)*.

Finally results on interacting variables revealed a combination of difficulties with ‘demarcation’ and high ‘quantitative demands’ *(p < 0.000)* as significantly associated with a severe work-private life conflict and also that the combination of ‘demands to hide emotions’ and ‘number of years in the current position’ is a relevant predictor for ‘work-private life conflict’ *(p < 0.000).*

### Quality of leadership

Most participating health professionals (85%) had no management responsibilities, 10% of them worked at a lower-management level (e.g. team leader, ward manager), 4% in the middle-management level (e.g. divisional manager, senior or leading physician) and 1% in an upper-management level (e.g. directors, hospital director, clinic director).

Participating health professionals were also asked to assess the leadership qualities of their direct line manager (in terms of promoting development opportunities, job satisfaction, good work planning and conflict management). Most health professionals rated the leadership qualities of their superior as good to a ‘large or very large extent’ (nurses and midwives = 69.4%, physicians = 64.8%, medical-technical professionals = 61.9%, medical-therapeutic professionals = 74.5%). However, another 21.4% of the nurses and midwives, 24.4% of the physicians, 28.3% the medical-technical professionals and 18.6% of the medical-therapeutic professionals rated the leadership qualities of their superior as ‘poor or very poor‘.

Results from the final multilevel model on ‘quality of leadership’ are presented in Table 4 (predictors explained 60.7% of the variance). Perceived ‘social support’ at work (from colleagues as well as from their line manager) was found to be strongly related to how health professionals rated the leadership qualities of their direct line manager *(B = 0.61, p = 0.000).* The perceived ‘reward’ (e.g. recognition and appreciation) from the health professional’s line manager was also a relevant predictor for the perceived ‘quality of leadership’ of their line manager *(B = 0.41, p = 0.000)*. The scale on ‘emotional demands’ at work indicates contrasting results: higher ‘emotional demands’ at work was associated with a better-rated ‘quality of leadership’ of their line manager *(B = 0.41, p = 0.000).* However, higher ‘quantitative demands’ at work predicted a lower-rated ‘quality of leadership’ for the health professionals’ line manager *(B = -0.27, p = 0.000).* Moreover, a higher ‘predictability’ of work (e.g. being informed in advance about important decisions, changes or plans) *(B = 0.25, p = 0.000)* as well as fewer ‘social relations at work’ *(B = -0.16, p = 0.000)* were also relevant predictors for ‘quality of leadership’ among health professionals. Finally, results on interacting variables revealed that a combination of health professionals’ perceived ‘social support’ and reward’ at work was significantly associated with how they rated the ‘quality of leadership’ of their superiors *(p < 0.000).*

**Table 4:** Results of multiple regression analysis on ‘quality of leadership’

coefficients	estimate (B)	std. estimate (β)	std. error^a^	t-value^a^	*p*-value^a^
(Intercept)	−16.58	0.00	9.99	−1.66	0.100
Social support at work	0.61***	0.45	0.04	14.56	0.000
Rewards	0.41***	0.47	0.06	7.17	0.000
Predictability	0.25***	0.21	0.02	11.50	0.000
Bond with the company	−0.09	−0.08	0.09	−1.07	0.258
Feedback	−0.09	−0.08	0.07	−1.29	0.160
Social relations at work	−0.16***	−0.16	0.03	−5.33	0.000
Quantitative demands	−0.27***	−0.18	0.06	−4.71	0.000
Possibilities for development	0.07**	0.05	0.03	2.92	0.004
Emotional demands	0.41***	0.28	0.12	3.53	0.000
Unfair behaviour	−0.05***	−0.05	0.02	−3.27	0.000
Role clarity	0.15	0.10	0.11	1.37	0.156
Interactions – social support at work & rewards	0.00***	−0.46	0.00	−6.10	0.000
Interactions – rewards & social relations at work	0.00**	0.17	0.00	3.20	0.002
Interactions – emotional demands & role clarity	0.00**	−0.29	0.00	−2.99	0.002
Interactions – feedback & quantitative demands	0.00**	0.21	0.00	3.14	0.002
Interactions – bond with the company & role clarity	0.00**	0.25	0.00	2.76	0.006

^a^based on bootstrap, *p < 0.05, **p < 0.01, ***p < 0.001

## Discussion

Main results showed major differences regarding the extent of work-private life conflicts and associated factors among the health professionals’ different roles and professions. Findings of this study on the compatibility of work and private life among Swiss health professionals revealed that in some cases legal breaks and rest periods are not observed, and that overtime work seems to be relatively common, especially for physicians and nurses. Further results indicated that health professionals’ preferences (e.g. to work one specific shift only) and being able to influence their work schedule (no possibility to exchange shifts with colleagues), as well as having to work multiple shifts per weekend and/or working full-time were strongly related to a ‘work-private life conflict’.

The results of a systematic review [10] also revealed a strong relationship between a higher number of working hours and lower levels of work-life balance. In addition, employees’ influence over their work schedule was also related to their work-life balance [10], in which a stronger influence positively affected the compatibility of their work and private life. Therefore, the results of this study are in line with other study results, but provide additional findings on existing ‘role conflicts’, higher ‘demands for hiding emotions’, lower ‘meaning of work’, lower ‘bond with the organisation’, lack of ‘social community’ at work as well as ‘insecurity of working environment’ as being related to a higher work-private life conflict among health professionals in Swiss hospitals. It is particularly important for health care managers to know these related factors in order to be able to reduce these stressors effectively in daily practice.

Regarding possible interventions to increase health professionals’ influence on their work schedule or to decrease overtime and work hours, several intervention studies have already been conducted. In a study by Akerstedt, et al. [29] the minimizing reduction of working hours per shift with full pay and input of extra personnel resulted in positive social effects and increased employee well-being after 1 year. In addition, the study results of Kauffeld et al. [30] show that the implementation of a ‘flexible work-time design’ is strongly associated with a lower absenteeism level, higher work quality and increased employee satisfaction with work. Moreover, other findings [31] have shown significantly increased work-life balance and job satisfaction among nurses after the implementation of an ‘open-rota’ shift work system. As these and other findings demonstrate, developing strategies to increase health professionals’ influence and autonomy regarding their shift schedule would appear to be an effective strategy to better reconcile work and private life. Moreover, in view of the significant association between hours worked per week and work-private life conflicts, opportunities for part-time work should be improved as well [10], especially among physicians and nurses / midwives.

On the other hand, appropriate leadership in managing employees’ workload is also an important factor affecting health professional’s working hours and overtime [11]. Managerial leadership has a considerable influence on increasing or preventing work-related stress, and this is shown in the way managers behave towards their employees [11]. Most important findings on ‘quality of leadership’ have shown that perceived ‘social support at work’, ‘reward’ and ‘predictability’ of work are the mostly relevant associated factors among health professionals working in acute and rehabilitation hospitals.

There are already indications in the literature that ‘quality of leadership’ and ‘social support at work’ are linked [12–14]. As a previous review indicates, a supportive organisational culture enhances positive leadership styles and therefore has a positive effect on how health professionals experience leadership [32]. Moreover, there is evidence that a supportive work environment provided by leaders is also related to the perception of stress at work [33]. Perceived ‘reward’ at work is also a well-known influencing factor in the effort-reward imbalance model affecting employees’ well-being [17]. The findings of this study also provide information about the perceived ‘reward’ of health professionals being linked with their assessment of leadership qualities of their line manager.

However, our results also show that higher ‘emotional demands’ at work are associated with a better rated ‘quality of leadership’ of line managers. An interesting point related to this are the findings of Little et al. [34], who identified the ‘emotion management strategies’ (leader behaviours in managing employees’ negative emotions) used by the leader as being important for employees’ positive or negative perception of their leader. A possible explanation for our study results could therefore be that the way the line manager deals with emotional demands on the employees is decisive in how they perceive his/her leadership qualities. In any case, the results of this study imply that providing social support at work, ensuring reward and predictability of work as well as developing positive strategies to manage emotional demands seem to be important supervisor behaviours, independent of the chosen management style.

### Strengths and limitations

This study differs from many studies in that health professionals from various disciplines are included and the study sample is not limited to nurses or physicians only. This is particularly important because our results show managers which topics are equally relevant to all health professionals so that they can choose strategies that have a great leverage effect in their organisation. In addition, this study reveals results regarding the German- and French-speaking parts of Switzerland (most studies focus on the German-speaking part only) with a sufficiently large sample for each language part. In addition, the use of well established, valid and reliable scales supports the validity of these results. A further strength of the study is the sophisticated statistical analysis, in which explanatory variables were examined not only for their main effect, but also for possible interactions with other variables. This has the advantage that important relationships between the explanatory variables can also be uncovered; e.g. the interaction of the scale on ‘demarcation’ and ‘quantitative demands’ was a more relevant predictor on ‘work-private life conflict’ than the main effect of ‘demarcation’ separately.

There are, however, also limitations. One of these is the study’s cross-sectional design, which means that causal conclusions cannot be drawn from our results. This is particularly relevant, since longitudinal data would be necessary to confirm our data of relevant predictors of health professionals’ work-private life conflicts and the perceived quality of leadership of line managers. Also, the results of our study are limited to our selection of independent variables available from the STRAIN questionnaire within the regression models. Using additional questionnaires or items (e.g. the effort-reward-imbalance questionnaire or scales to assess the prevailing culture at work) to predict work-private life conflicts and leadership qualities, could have provided additional information. As a consequence, there may be further important influencing factors for health professionals’ work-private life conflicts and perceived quality of leadership that are not included in our results. Furthermore, results presented in this study are based on data from the Swiss healthcare sector and thus influenced by Swiss labour law (e.g. regarding working hours). Therefore, results from other countries, especially regarding prevalence results on work-private life conflicts, might differ. In addition, participation was on a voluntary basis for health care organisations as well as for all health professionals and, therefore, a selection bias is possible. This could mean that some health professionals who were more affected by stress at work may not have participated (e.g. for time reasons) and may be underrepresented in our results (i.e., prevalence results are lower than in the total population of health professionals). On the other hand, it is also possible that more health professionals suffering from severe job dissatisfaction may have filled in the questionnaire and may be overrepresented in our study sample (i.e., prevalence results are higher than in the total population of health professionals).

## Conclusions

Optimal compatibility of work and private life as well as competent leadership qualities among line managers are key issues in ensuring that health professionals remain in their profession. Managerial support, appropriate reward and greater predictability of work and plans for the future were determined to be particularly important in improving the quality of leadership among health professional leaders. Moreover, the findings of this study imply that it is important to consider health professionals’ preferences and to increase their influence and autonomy regarding their working schedule to improve the balance of their work and private life. It is not only important to take greater account of personal preferences when planning shifts, but also to provide more flexible work-time designs that make this possible, for example, by allowing the exchange of shifts if necessary. More flexible work-time designs are increasingly in demand in hospitals, especially in order to retain existing health care staff in their careers long-term. Therefore, further studies to develop and test flexible work-time designs are essential, especially in view of the immense demands of work in the health sector.

Next to this, competent leaders are also needed to help achieve a better balance in health professionals’ work and private life, to increase adherence to legal breaks and rest periods and to optimize the management of workload and overtime. Health professional leaders play a key role in alleviating stress at work and are in a position to increase or decrease the level of stress among their employees through the way they behave. Therefore, further research is needed to better support managers in preventing and reducing imbalances in the work and private lives of their employees as well to further develop good leadership qualities in line managers.

## Data Availability

The raw dataset analysed in the current study is available from the corresponding author on reasonable request.

## Abbreviations

AIC: Akaike Information Criterion
BIC: Bayesian Information Criterion
COPSOQ: Copenhagen Psychosocial Questionnaire
EWCS: European Working Conditions Survey
NEXT: Nurses’ early exit study
OSLO: Oslo social support scale
STRAIN: Work-related stress among health professionals in Switzerland
